# Magnetic Au-Ag-γ-Fe_2_O_3_/rGO Nanocomposites as an Efficient Catalyst for the Reduction of 4-Nitrophenol

**DOI:** 10.3390/nano8110877

**Published:** 2018-10-25

**Authors:** Guangyu Lei, Jingwen Ma, Zhen Li, Xiaobin Fan, Wenchao Peng, Guoliang Zhang, Fengbao Zhang, Yang Li

**Affiliations:** Lab of Advanced Nano-structures & Transfer Processes, Department of Chemical Engineering, Tianjin University, Tianjin 300354, China; guangyulei@tju.edu.cn (G.L.); jingwenma@cup.edu.cn (J.M.); lizhen@tust.edu.cn (Z.L.); xiaobinfan@tju.edu.cn (X.F.); wenchao.peng@tju.edu.cn (W.P.); zhangguoliang@tju.edu.cn (G.Z.)

**Keywords:** graphene oxide, Au-Ag-γ-Fe_2_O_3_/rGO nanocomposites, 4-NP, magnetism

## Abstract

In this paper, a facile route has been developed to prepare magnetic trimetallic Au-Ag-γ-Fe_2_O_3_/rGO nanocomposites. The impact of the preparation method (the intensity of reductant) on the catalytic performance was investigated. The nanocomposites were characterized by transmission electron microscopy (TEM), X-ray diffraction (XRD) and X-ray photoelectron spectroscopy (XPS). The prepared nanocomposites show fine catalytic activity towards the reduction reaction of 4-nitrophenol (4-NP). The nanocomposites also have superparamagnetism at room temperature, which can be easily separated from the reaction systems by applying an external magnetic field.

## 1. Introduction

Over the years, nanoparticles have been widely utilized in the fields of energy, biomedicine and environmental pollution prevention [1,2]. Compared with bulk materials [3,4], noble metal nanoparticles are applied more broadly to heterogeneous catalytic processes because of their distinctive physicochemical properties, such as large specific surface area and high Fermi potential. Polymetallic nanoparticles show especially enhanced catalytic properties that are caused by synergistic effects [5]. For instance, Au-Pt nanoparticles perform excellently in the oxidation of methanol [6], Au-Pd nanoparticles have been used as catalysts for the oxidation of TMB [7] and Au–Ag nanocrystals have been used in hydrogen evolution reactions [8]. However, the aggregation of nanoparticles may result in a decrease of catalytic activity. To maximize the activity of noble metal nanoparticles, a suitable support is necessary to ensure dispersion. Graphene is a promising candidate which has attracted great attention due to its excellent chemical and physical properties, such as large specific surface area [9], outstanding mechanical strength [10,11] and unique thermal [12] and electronic [13] properties. As a derivative of graphene, graphene oxide (GO) is a common choice of support [14]. This derivative, similar to graphene, has unique properties and is convenient to prepare. It should be noted that nanocatalysts are difficult to use in continuous flow systems due to their tiny size and high stability in reaction systems. To solve this problem, nanocatalysts are combined with magnetic nanoparticles, such as Fe_3_O_4_ and γ-Fe_2_O_3_ [15,16]. These hybrids are easy to retrieve from the reaction system via a magnetic field due to their magnetic properties. Magnetic separator technology has been widely used in various fields and is quite desirable in the corresponding industries because it can overcome the disadvantages of batch operation and other issues of filtration or centrifugation.

In this paper, γ-Fe_2_O_3_, Au and Ag nanoparticles were supported by partially reduced graphene oxide (Au-Ag-γ-Fe_2_O_3_/rGO). With well-dispersed nanocatalysts and a synergistic effect between Au and Ag, the prepared composites showed superior catalytic activity in the reduction reaction of 4-nitrophenol (4-NP). Moreover, the hybrids could be easily separated from the reaction systems by simply applying an external magnetic field at room temperature with the help of their superparamagnetism. The multiplexing experiments also demonstrated remarkable reusability and stability.

## 2. Materials and Methods

### 2.1. Materials

The graphite power was from Huadong Graphite Factory, Qingdao, Shandong, China; the silver nitrate was from Heowns Biochem Technologies LLC, Tianjin, China; the sodium borohydride was from Aladdin, Shanghai, China; all other reagents were from Tianjin Guangfu Fine Chemical Research Institute, Tianjin, China. All reagents were used as received.

### 2.2. Preparation of Au-Ag-γ-Fe_2_O_3_/rGO Nanocomposites

Graphene oxide (GO) was prepared from natural graphite by the modified Hummers method [17]. The γ-Fe_2_O_3_/rGO nanocomposites were then synthesized by a mixture of GO and FeSO_4_. In this process, 0.1 g of GO were dispersed in 100.0 mL of distilled water by ultra-sonication to form a homogeneous dispersion. The mixture was heated to 80 °C with magnetic stirring. Then, 0.25 g of FeSO_4_·7H_2_O were dispersed in 10.0 mL of distilled water, with 3 mL of ammonium hydroxide (25%) added subsequently. After magnetic stirring for another hour, the mixture was cooled to room temperature and washed several times with distilled water to obtain black product (γ-Fe_2_O_3_/rGO). The Au-Ag-γ-Fe_2_O_3_/rGO nanocomposites were then synthesized from the mixture of γ-Fe_2_O_3_/rGO, AgNO_3_ and HAuCl_4_. In this process, 15.0 mg of γ-Fe_2_O_3_/rGO and trisodium citrate were dispersed in 20.0 mL of distilled water. The mixture was heated to 100 °C in an oil bath before 0.25 mL of HAuCl_4_ (20 mM), 0.5 mL of AgNO_3_ (10 mM) and 1 mL of ascorbic acid (AA, 0.1 M) were added. After stirring for 3.5 h at 100 °C, the mixture was cooled to room temperature and washed with distilled water several times to obtain black product (AA-Au-Ag-γ-Fe_2_O_3_/rGO). For comparative purposes, the same preparation conditions were used to prepare AA-Ag-γ-Fe_2_O_3_/rGO, AA-Au-γ-Fe_2_O_3_/rGO and SDS-Au-Ag-γ-Fe_2_O_3_/rGO using sodium dodecyl sulfate (SDS) as the reducing and dispersing agent instead of AA. Please refer to the Supplementary Materials for the detailed preparation process.

### 2.3. Characterization

The morphology and dispersion of the as-prepared Au-Ag-γ-Fe_2_O_3_/rGO nanocomposites were characterized by high-resolution transmission electron microscopy (HRTEM). The HRTEM measurement was performed using a Philips Tecnai G2F20 microscope (Tianjin, China) at 200 kV. The X-ray diffraction (XRD) was carried out using a Bruker-Nonius D8 Focus diffractometer (Tianjin, China) with Cu-Kα radiation (λ = 0.15418 nm) at 40 kV and 100 mA scanning in the range of 2θ = 15–90°. X-ray photoelectron spectroscopy (XPS) was conducted on an X-ray photoelectron spectrometer (Perkin- Elmer, PHI 1600 spectrometer, Beijing, China). The catalytic activity of Au-Ag-γ-Fe_2_O_3_/rGO nanocomposites was measured by UV absorption spectra on an UV-2802H system (Tianjin, China) with temperature control. The degradation pathways were determined by detecting intermediates using an LC-TOF mass spectrometer (Tianjin, China) with electro spray ionization (ESI) and C18 column (150 mm × 2 mm) (30:70v/v methanol: water as mobile phase; 2% formic acid; injection volume 20 µL).

### 2.4. Catalytic Reduction of 4-NP by Au-Ag-γ-Fe_2_O_3_/rGO Nanocomposites

The reduction reaction of 4-NP was chosen as a model reaction for investigating the catalytic activity of the as-prepared Au-Ag-γ-Fe_2_O_3_/rGO nanocomposites. Typically, 2.8 mL of 4-NP aqueous solution (1.0 × 10^–4^ M) was added into a quartz cuvette at room temperature; then, 30 μg of catalyst and 0.2 mL of freshly prepared NaBH_4_ aqueous solution (0.15 M) were added subsequently. The concentration of 4-NP was monitored by observing the UV−visible (UV-VIS) absorption spectra from 250 to 550 nm. To investigate the recyclability of the catalyst, the catalyst was recovered by magnetic separation and washed with distilled water before being added to another freshly prepared, mixed solution for the next round of catalysis.

## 3. Results and Discussion

### 3.1. Synthesis of Au-Ag-γ-Fe_2_O_3_/rGO Nanocomposites

**Figure 1:**
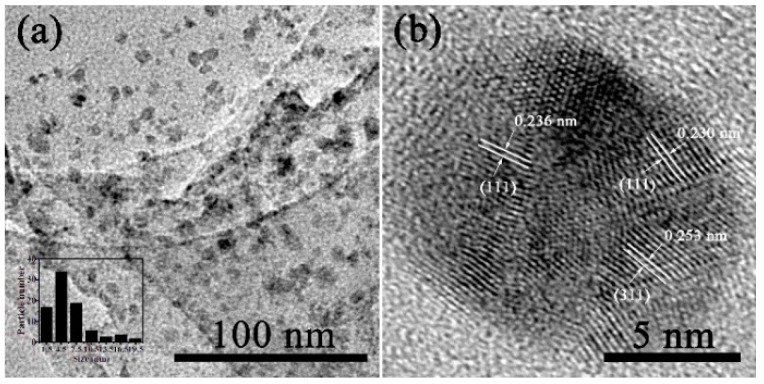
TEM image (**a**) (inset is the corresponding size distribution) and (**b**) high-resolution transmission electron microscopy (HRTEM) image of Au-Ag-γ-Fe_2_O_3_/rGO.

The TEM images (Figure 1a) show that spherical nanoparticles are dispersed densely on the surface of rGO with no free nanoparticles visible, confirming the decoration of the noble metal nanoparticles on graphene. The average size of the nanocomposites is around 5.3 nm. The HRTEM image (Figure 1b) shows the typical crystal lattice of trimetallic nanocomposites with a lattice spacing of 0.236 nm, which corresponds to the (111) plane of Ag [18,19], while the lattice spacing of 0.230 nm and 0.253 nm are representative of the (111) plane of Au [20] and the (311) plane of γ-Fe_2_O_3_ [21], respectively. The morphology of the comparative nanocomposite SDS-Au-Ag-γ-Fe_2_O_3_/rGO was also investigated by TEM to note the differences in morphology and dispersion (Figure S1, Supporting Information). The metal nanoparticles prepared with SDS as the reducing agent have obvious agglomeration, while those prepared with AA as the reductant were distributed evenly. Such results indicate that the stronger the reducing agent, the faster the nucleation and the more homogeneous the nanoparticles become.

**Figure 2:**
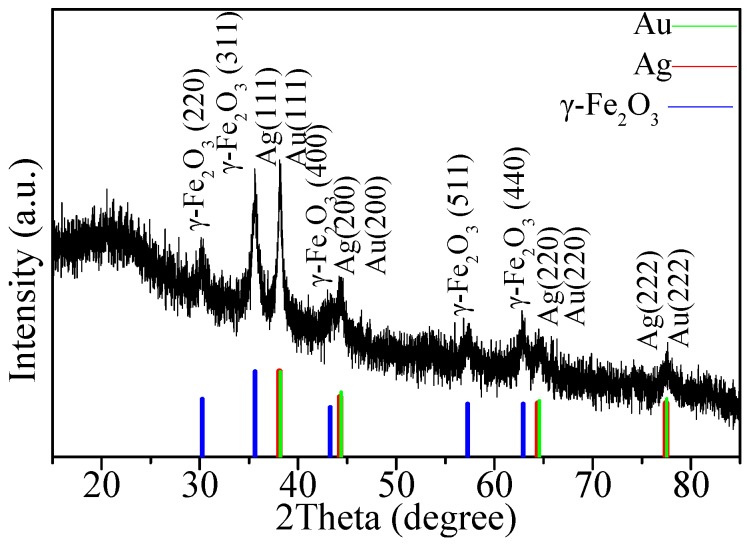
XRD pattern of Au-Ag-γ-Fe_2_O_3_/rGO.

The XRD patterns of the hybrids revealed the face-centered cubic (fcc) structure of the nanoparticles with distinguished peaks, especially the (311) planes of γ-Fe_2_O_3_ and the (111) planes of Ag and Au (Figure 2). The peaks at 2θ values of 30.2° (220), 35.6° (311), 43.3° (400), 57.3° (511) and 62.9° (440) were consistent with the standard XRD data of γ-Fe_2_O_3_. The other four peaks of the nanocomposites at 38.1° (111), 44.3° (200), 64.5° (220) and 77.5° (222) can be assigned to Ag and Au. Since the diffraction peaks of Ag and Au were very close to each other, it was difficult to distinguish between them.

**Figure 3:**
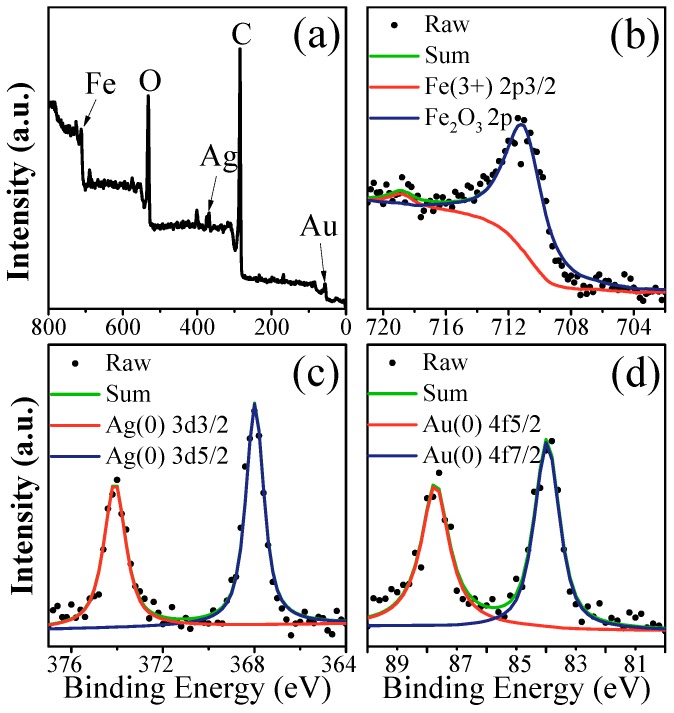
XPS spectra of (**a**) full scan, (**b**) Fe 2p, (**c**) Ag 3d doublet and (**d**) Au 4f doublet of Au-Ag-γ-Fe_2_O_3_/rGO, respectively.

XPS is an effective method for investigating the elemental composition of materials. The data have been adjusted based on the binding energy of C 1s to 284.6 eV. Figure 3a shows the XPS spectrum of Au-Ag-γ-Fe_2_O_3_/rGO nanocomposites, from which the surface atomic ratio of Fe to Au to Ag was found to be 2.05:0.11:0.29. The value was not quite to the atomic ratio in the initial metal precursors (9:1:1), which might have been due to the fact that XPS analysis only detects the surface properties of the catalysts. Figure 3b shows the XPS spectrum of the Fe 2p (FeIII2p_3/2_ and Fe_2_O_3_2p) for Fe. There was an apparent peak at 711.0 eV and a suspected satellite peak at 718.8 eV. It was reported that the Fe_2_O_3_ peak [22] at 711.0 eV with a satellite peak at around 719.0 eV represented Fe^3+^ ions. These results support the formation of γ-Fe_2_O_3_ on graphene surfaces. Figure 3c,d show the typical XPS spectra of Ag (3d_5/2_ and 3d_3/2_) (368.0 and 374.0 eV) and Au (4f_7/2_ and 4f_5/2_) (84.0 and 87.8 eV) in Au-Ag-γ-Fe_2_O_3_/rGO nanocomposites. Hence, the formation of Ag and Au on the surface of graphene is further confirmed by XPS analysis. Arising from the electron transfer of GO nanosheets to metal nanoparticles, these peaks shift to slightly lower binding energies. Chemical and structural changes of GO after the fact were also investigated by XPS analysis (Figure S2, Supporting Information) and showed evidence of the removal of negatively charged oxide functional groups, which confirms the reduction of GO to rGO.

### 3.2. Catalytic Reaction of 4-NP by Au-Ag-γ-Fe_2_O_3_/rGO Nanocomposites

The catalytic performance of the Au-Ag-γ-Fe_2_O_3_/rGO nanocomposites was evaluated by the reduction reaction of 4-nitrophenol (4-NP). Since the reduction product 4-aminophenol (4-AP) is wildly needed in many fields [23], it has become essential to establish a low energy consumption and environmentally friendly process for the reduction reaction. Hence, the reaction was carried out at room temperature with the addition of NaBH_4_ in the aqueous phase. The 4-NP aqueous solution showed maximum absorption at 377 nm. After the addition of NaBH_4_ (pH > 12.0), the absorption peak of 4-NP experienced a red shift due to the formation of a 4-nitrophenolate ion; at 400 nm the color changed from light yellow to deep yellow [24]. The extent of the conversion of 4-NP to 4-AP can be easily monitored by tracking the changes in the absorbance peak at 400 nm since only one product forms (4-AP, 295 nm) [25]. After the reaction, the catalysts were recovered by an external magnet.

In the absence of catalysts, no decrease in the absorbance peak at 400 nm was observed after several hours, even when NaBH_4_ and 4-NP were added into the system. Circumstances did not change with either the addition of GO or γ-Fe_2_O_3_/rGO (Figure S3a, Supporting Information). When only 30 µg of the Au-Ag-γ-Fe_2_O_3_/rGO nanocomposites were added, the reduction started quickly (Figure S3b, Supporting Information). In time, the yellow color of 4-NP under alkali conditions was completely bleached as the absorbance intensity of the UV-VIS spectroscopy decreased. To further confirm the completion of the reduction reaction, LC-MS analysis of initial and final reaction mixtures was carried out. The results are given in Figure S4a,b indicating a clear and clean conversion of nitrophenol into aminophenol. These results prove that the catalytic activity originates from the Au and Ag of Au-Ag-γ-Fe_2_O_3_/rGO. The 4-NP molecules were adsorbed onto the rGO sheets once the catalysts were added because of the hydrophobic interaction that caused improved partial concentration; then, the Au-Ag alloy nanoparticles on the nanocomposites served as catalysts to transfer electrons from BH_4_^−^ ions to the 4-NP. Owing to the large specific surface area and the excellent electronic mobility of the rGO, the catalysis performance greatly improved.

**Figure 4:**
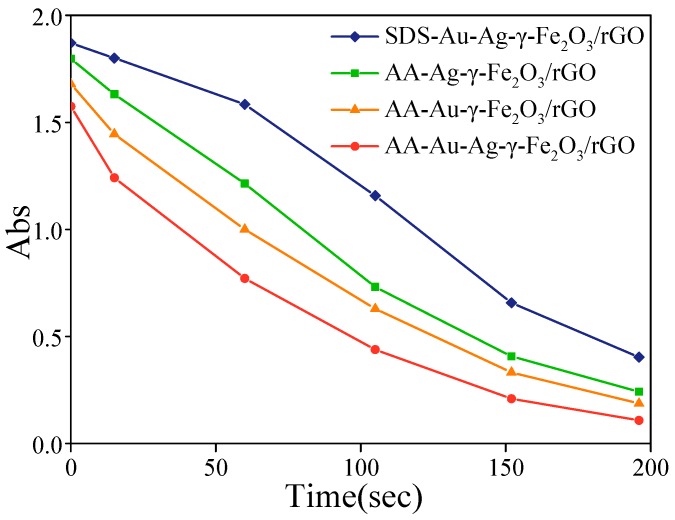
Time-dependent absorbance changes at 400 nm in the presence of SDS-Au-Ag-γ-Fe_2_O_3_/rGO, AA-Ag-γ-Fe_2_O_3_/rGO, AA-Au-γ-Fe_2_O_3_/rGO and AA-Au-Ag-γ-Fe_2_O_3_/rGO.

Figure 4 shows the absorbance versus time plots at 400 nm in the presence of SDS-Au-Ag-γ-Fe_2_O_3_/rGO, AA-Ag-γ-Fe_2_O_3_/rGO, AA-Au-γ-Fe_2_O_3_/rGO and AA-Au-Ag-γ-Fe_2_O_3_/rGO nanocomposites, respectively. Since the initial concentration of BH_4_^−^ was much higher than that of 4-NP and remained mainly constant during the reaction, a pseudo-first-order reaction kinetic has been applied for evaluation of the catalytic rate. The kinetic equation can be written as:

ln(*C*/*C*_0_) = ln(*A*/*A*_0_) = −*kt*

where *C*_0_ is the initial concentration of 4-NP; *C* is the concentration of 4-NP at any time; *A_0_* is the absorbance at 400 nm replacing NaBH_4_ by the same amount of NaOH; *A* is the absorbance at 400 nm at any time; *t* is the time after the addition of NaBH_4_; *k* is the rate constant. The corresponding pseudo-first-order rate constants for SDS-Au-Ag-γ-Fe_2_O_3_/rGO, AA-Ag-γ-Fe_2_O_3_/rGO, AA-Au-γ-Fe_2_O_3_/rGO and AA-Au-Ag-γ-Fe_2_O_3_/rGO were found to be 0.0068 s^−1^, 0.0097 s^−1^, 0.0107 s^−1^ and 0.0133 s^−1^, respectively. These results indicate that the prepared AA-Au-Ag-γ-Fe_2_O_3_/rGO nanocomposites have high catalytic efficiency in relation to the 4-NP reduction reaction. It will be significant to compare the catalytic performance of this preparation of nanocomposites with previously reported analogous catalysts. It can be seen from Table 1 that Au-Ag-γ-Fe_2_O_3_/rGO nanocomposites show decent performance in the reduction of 4-NP.

Since the work function of Ag is lower than that of Au, electrons tend to move from the Ag region to the Au region [33]. These surplus electrons inside the Au region improved the catalytic performance toward the 4-NP reduction reaction [27]. It should be noted that the nanocomposites using AA as the reducing agent showed higher catalytic activity compared with those using SDS as the reductant. A possible reason for this is that the reducing ability of AA was stronger than that of SDS. When using a weaker reductant, metal growth speed is relatively slow, resulting in larger metal nanoparticles. When the initial amount of precursor is fixed, larger particles have less specific surface area, leading to poorer catalytic performance. This is consistent with the TEM results mentioned above.

Reusability is an important property to consider when evaluating the performance of a catalyst. The γ-Fe_2_O_3_ has superior superparamagnetism at room temperature, which could debase the immense complexity of the separate operations. To investigate the reusability of Au-Ag-γ-Fe_2_O_3_/rGO nanocomposites, the catalysts were carefully collected from the reaction solution by a magnet after the reduction reaction and used for subsequently repeated experiments. The catalysts exhibited similar catalytic performance on the conversion of 4-NP, within the same reaction time, even after running for five cycles (Figure S5, Supporting Information). The fifth reuse rate of reaction remained at more than 95% of that of the first time, indicating the outstanding recyclable performance of the catalyst. It was also essential to possess superparamagnetism when collecting and reusing the catalysts. The nanocomposites could be successfully gathered in seconds with a magnetic field, allowing the catalysts to be rapidly separated and recycled.

## 4. Conclusions

In summary, a simple method was employed to prepare novel and reusable Au-Ag-γ-Fe_2_O_3_/rGO nanocomposites. Moreover, the impact of the preparation method indicated that using strong reductant during the preparation process tended to form tinier particles. The prepared nanocomposites were successfully applied in the catalytic reduction of 4-NP with excellent catalytic activity and reusability and could be easily separated from the reaction mixture because of the introduction of the magnetic nanoparticles. Such magnetic nanocomposites are expected to be employed in many other fields and for industrial applications.

## Figures and Tables

**Table 1:** Comparison of the nanocatalysts for the reduction of 4-NP.

Catalyst	Catalyst Usage Amount (mg)	Concentration of 4-NP (M)	Rate Constant k (s^−1^)	Normalized Rate Constant k * (s^−1^ mg^−1^) ^a^	References
Au-Ag-γ-Fe_2_O_3_/rGO	0.03	1.0 × 10^−4^	0.0133	0.443	Present work
Bi-Fe_3_O_4_@RGO	0.5	1.54 × 10^−3^	0.00808	0.016	[26]
AuAg−G (OB)	0.03	1.0 × 10^−4^	0.0089	0.297	[27]
Fe_3_O_4_/Au	0.05	6.7 × 10^−5^	0.00728	0.146	[28]
GO/Ag−Fe_3_O_4_	0.09	0.01	0.0142	0.158	[29]
Ag-Fe_3_O_4_/RGO	4.0	1.0 × 10^−4^	0.044	0.011	[30]
Pd/Fe_3_O_4_@SiO_2_@KCC-1	0.025	1.0 × 10^−4^	0.0196	0.784	[31]
Au/HNTs/Fe_3_O_4_	50	5.0 × 10^−3^	0.021	4.2 × 10^−4^	[32]

^a^ k *: rate constant normalized to the catalyst usage amount. Data were given or calculated in the respective papers.

## Supplementary Materials

The following are available online at http://www.mdpi.com/2079-4991/8/11/877/s1, the detailed preparation process of SDS-Au-Ag-γ-Fe_2_O_3_/rGO, AA-Au-γ-Fe_2_O_3_/rGO and AA-Ag-γ-Fe_2_O_3_/rGO, Figure S1: TEM image of SDS-γ-Fe_2_O_3_-Au-Ag/rGO, Figure S2: XPS spectra of γ-Fe_2_O_3_-Au-Ag/rGO, Figure S3: UV-vis absorption spectra of the reduction 4-NP by NaBH_4_ in the presence of (a) AA-γ-Fe_2_O_3_/rGO, (b) AA-γ-Fe_2_O_3_-Au-Ag/rGO, Figure S4: LC-MS spectrum for (**a**) 4-NP (**b**) for 4-AP (final product), Figure S5: UV- vis absorption spectra of the reduction 4-NP by NaBH_4_ in the presence of (**a**) AA-γ-Fe_2_O_3_-Au-Ag/rGO (**b**) AA-γ-Fe_2_O_3_-Au-Ag/rGO after running for five cycles.

## References

[1] Kelly K.L., Coronado E., Zhao L.L., Schatz G.C. (2003). The optical properties of metal nanoparticles: The influence of size, shape, and dielectric environment. J. Phys. Chem. B.

[2] Arico A.S., Bruce P., Scrosati B., Tarascon J.M., Van Schalkwijk W. (2005). Nanostructured materials for advanced energy conversion and storage devices. Nat. Mater..

[3] Daniel M.C., Astruc D. (2004). Gold nanoparticles: Assembly, supramolecular chemistry, quantum-size-related properties, and applications toward biology, catalysis, and nanotechnology. Chem. Rev..

[4] Mohamed M.M., Al-Sharif M.S. (2012). One pot synthesis of silver nanoparticles supported on TiO2 using hybrid polymers as template and its efficient catalysis for the reduction of 4-nitrophenol. Mater. Chem. Phys..

[5] Gilroy K.D., Ruditskiy A., Peng H.C., Qin D., Xia Y.N. (2016). Bimetallic Nanocrystals: Syntheses, Properties, and Applications. Chem. Rev..

[6] Ma J., Wang J., Zhang G., Fan X., Zhang G., Zhang F., Li Y. (2015). Deoxyribonucleic acid-directed growth of well dispersed nickel-palladium-platinum nanoclusters on graphene as an efficient catalyst for ethanol electrooxidation. J. Power Sources.

[7] Chen H., Li Y., Zhang F., Zhang G., Fan X. (2011). Graphene supported Au-Pd bimetallic nanoparticles with core-shell structures and superior peroxidase-like activities. J. Mat. Chem..

[8] Qin Y., Dai X., Zhang X., Huang X., Sun H., Gao D., Yu Y., Zhang P., Jiang Y., Zhuo H. (2016). Microwave-assisted synthesis of multiply-twinned Au-Ag nanocrystals on reduced graphene oxide for high catalytic performance towards hydrogen evolution reaction. J. Phys. Chem. A.

[9] Chae H.K., Siberio-Perez D.Y., Kim J., Go Y., Eddaoudi M., Matzger A.J., O’Keeffe M., Yaghi O.M. (2004). A route to high surface area, porosity and inclusion of large molecules in crystals. Nature.

[10] Meyer J.C., Geim A.K., Katsnelson M.I., Novoselov K.S., Booth T.J., Roth S. (2007). The structure of suspended graphene sheets. Nature.

[11] Lee C., Wei X.D., Kysar J.W., Hone J. (2008). Measurement of the elastic properties and intrinsic strength of monolayer graphene. Science.

[12] Balandin A.A., Ghosh S., Bao W.Z., Calizo I., Teweldebrhan D., Miao F., Lau C.N. (2008). Superior thermal conductivity of single-layer graphene. Nano Lett..

[13] Novoselov K.S., Geim A.K., Morozov S.V., Jiang D., Katsnelson M.I., Grigorieva I.V., Dubonos S.V., Firsov A.A. (2005). Two-dimensional gas of massless Dirac fermions in graphene. Nature.

[14] Su C.L., Loh K.P. (2013). Carbocatalysts: Graphene Oxide and Its Derivatives. Accounts Chem. Res..

[15] Bartolome L., Imran M., Lee K.G., Sangalang A., Ahn J.K., Kim D.H. (2014). Superparamagnetic gamma-Fe2O3 nanoparticles as an easily recoverable catalyst for the chemical recycling of PET. Green Chem..

[16] Paul B., Purkayastha D.D., Dhar S.S., Das S., Haldar S. (2016). Facile one-pot strategy to prepare Ag/Fe2O3 decorated reduced graphene oxide nanocomposite and its catalytic application in chemoselective reduction of nitroarenes. J. Alloys Compd..

[17] Hummers W.S., Offeman R.E. (1958). Preparation of Graphitic Oxide. J. Am. Chem. Soc..

[18] Vrijmoeth J., van der Vegt H.A., Meyer J.A., Vlieg E., Behm R.J. (1994). Surfactant-induced layer-by-layer growth of Ag on Ag(111): Origins and side effects. Phys. Rev. Lett..

[19] Van der Vegt H.A., van Pinxteren H.M., Lohmeier M., Vlieg E., Thornton J.M. (1992). Surfactant-induced layer-by-layer growth of Ag on Ag(111). Phys. Rev. Lett..

[20] Majimel J., Lamirand-Majimel M., Moog I., Feral-Martin C., Treguer-Delapierre M. (2009). Size-Dependent Stability of Supported Gold Nanostructures onto Ceria: An HRTEM Study. J. Phys. Chem. C.

[21] Babay S., Mhiri T., Toumi M. (2015). Synthesis, structural and spectroscopic characterizations of maghemite gamma-Fe2O3 prepared by one-step coprecipitation route. J. Mol. Struct..

[22] Shwan S., Jansson J., Olsson L., Skoglundh M. (2014). Chemical deactivation of Fe-BEA as NH3-SCR catalyst-Effect of phosphorous. Appl. Catal. B-Environ..

[23] Saha S., Pal A., Kundu S., Basu S., Pal T. (2010). Photochemical Green Synthesis of Calcium-Alginate-Stabilized Ag and Au Nanoparticles and Their Catalytic Application to 4-Nitrophenol Reduction. Langmuir.

[24] Pradhan N., Pal A., Pal T. (2001). Catalytic Reduction of Aromatic Nitro Compounds by Coinage Metal Nanoparticles. Langmuir.

[25] Mandlimath T.R., Gopal B. (2011). Catalytic activity of first row transition metal oxides in the conversion of p-nitrophenol to p-anninophenol. J. Mol. Catal. A-Chem..

[26] Wang X., Xia F., Li X., Xu X., Wang H., Yang N., Gao J. (2015). Fabrication of Bi-Fe3O4@RGO hybrids and their catalytic performance for the reduction of 4-nitrophenol. J. Nanopart. Res..

[27] Chen H., Fan X., Ma J., Zhang G., Zhang F., Li Y. (2014). Green Route for Microwave-Assisted Preparation of AuAg-Alloy-Decorated Graphene Hybrids with Superior 4-NP Reduction Catalytic Activity. Ind. Eng. Chem. Res..

[28] Yan F., Sun R. (2014). Facile synthesis of bifunctional Fe3O4/Au nanocomposite and their application in catalytic reduction of 4-nitrophenol. Mater. Res. Bull..

[29] Qu J.-C., Ren C.-L., Dong Y.-L., Chang Y.-P., Zhou M., Chen X.-G. (2012). Facile synthesis of multifunctional graphene oxide/AgNPs-Fe3O4 nanocomposite: A highly integrated catalysts. Chem. Eng. J..

[30] Joshi M.K., Pant H.R., Kim H.J., Kim J.H., Kim C.S. (2014). One-pot synthesis of Ag-iron oxide/reduced graphene oxide nanocomposite via hydrothermal treatment. Colloids Surf. A.

[31] Le X., Dong Z., Liu Y., Jin Z., Thanh-Do H., Minhdong L., Ma J. (2014). Palladium nanoparticles immobilized on core-shell magnetic fibers as a highly efficient and recyclable heterogeneous catalyst for the reduction of 4-nitrophenol and Suzuki coupling reactions. J. Mater. Chem. A.

[32] Mu B., Zhang W., Wang A. (2014). Facile fabrication of superparamagnetic coaxial gold/halloysite nanotubes/Fe3O4 nanocomposites with excellent catalytic property for 4-nitrophenol reduction. J. Mater. Sci..

[33] Wilson O.M., Scott R.W.J., Garcia-Martinez J.C., Crooks R.M. (2005). Synthesis, characterization, and structure-selective extraction of 1-3-nm diameter AuAg dendrimer-encapsulated bimetallic nanoparticles. J. Am. Chem. Soc..

