# Supplementary material for: Magnetic Au-Ag-γ-Fe2O3/rGO Nanocomposites as an Efficient Catalyst for the Reduction of 4-Nitrophenol
Source: Nanomaterials (Basel). 2018 Oct 25;8(11):877. doi: 10.3390/nano8110877 (PMC6266811; doi:10.3390/nano8110877)
Supplement: Supplementary file 1 [file nanomaterials-08-00877-s001.pdf]

Supplementary Materials

Magnetic Au-Ag-γ-Fe_2O_3/rGO Nanocomposites as an Efficient Catalyst for the Reduction of 4-Nitrophenol

Guangyu Lei, Jingwen Ma, Zhen Li, Xiaobin Fan, Wenchao Peng, Guoliang Zhang, Fengbao Zhang * and Yang Li *

Lab of Advanced Nano-structures & Transfer Processes, Department of Chemical Engineering, Tianjin University, Tianjin, 300354, China; guangyulei@tju.edu.cn (G.L.); jingwenma@cup.edu.cn (J.M.); lizhen@tust.edu.cn (Z.L.); xiaobinfan@tju.edu.cn (X.F.); wenchao.peng@tju.edu.cn (W.P.); zhangguoliang@tju.edu.cn (G.Z.)

* Correspondence: fbzhang@tju.edu.cn (F.Z.); liyang1895@tju.edu.cn (Y.L.); Tel.: +86-22-27890090 (F.Z. & Y.L.)

Methods

Preparation of SDS-Au-Ag-γ-Fe_2O_3/rGO Nanocomposites

SDS-Au-Ag-γ-Fe_2O_3/rGO nanocomposites was synthesized from mixture of γ-Fe_2O_3/rGO, $AgNO_3$ and $HAuCl_4$. In this process, 15.0 mg γ-Fe_2O_3/rGO and 28.7 mg of sodium dodecyl sulfate (SDS, 0.1 mmol) was dispersed in 20.0 mL of distilled water. The mixture was heated to 100 °C in oil bath, and then 0.25 mL of $HAuCl_4$ (20 mM) and 0.5 mL of $AgNO_3$ (10 mM) were added. After stirring for 3.5 h at 100 °C, the mixture was cooled to room temperature and washed with distilled water several times to obtain black product (SDS-Au-Ag-γ-Fe_2O_3/rGO).

Preparation of AA-Au-γ-Fe_2O_3/rGO Nanocomposites

AA-Au-γ-Fe_2O_3/rGO nanocomposites was synthesized from mixture of γ-Fe_2O_3/rGO, and $HAuCl_4$. In this process, 15.0 mg γ-Fe_2O_3/rGO and trisodium citrate was dispersed in 20.0 mL of distilled water. The mixture was heated to 100 °C in oil bath, and then 0.5 mL of $HAuCl_4$ (20 mM) and 1mL of ascorbic acid (AA, 0.1 M) were added. After stirring for 3.5 h at 100 °C, the mixture was cooled to room temperature and washed with distilled water several times to obtain black product (AA-Au-γ-Fe_2O_3/rGO).

Preparation of AA-Ag-γ-Fe_2O_3/rGO Nanocomposites

AA-Ag-γ-Fe_2O_3/rGO nanocomposites was synthesized from mixture of γ-Fe_2O_3/rGO, and $AgNO_3$. In this process, 15.0 mg γ-Fe_2O_3/rGO and trisodium citrate was dispersed in 20.0 mL of distilled water. The mixture was heated to 100 °C in oil bath, and then 1 mL of $AgNO_3$ (10 mM) and 1mL of ascorbic acid (AA, 0.1 M) were added. After stirring for 3.5 h at 100 °C, the mixture was cooled to room temperature and washed with distilled water several times to obtain black product (AA-Ag-γ-Fe_2O_3/rGO).

Figures

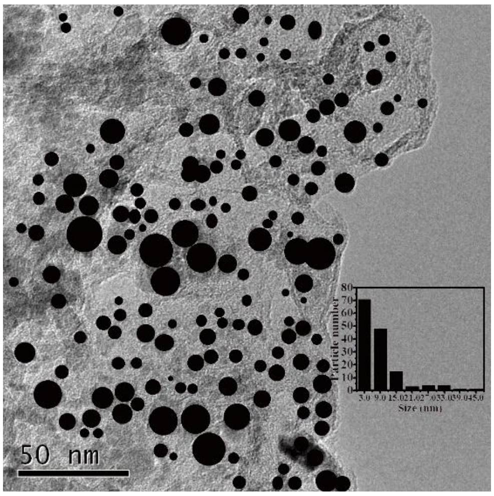

Figure S1. TEM image of SDS-γ-Fe_2O_3-Au-Ag/rGO (inset is the corresponding size distribution).

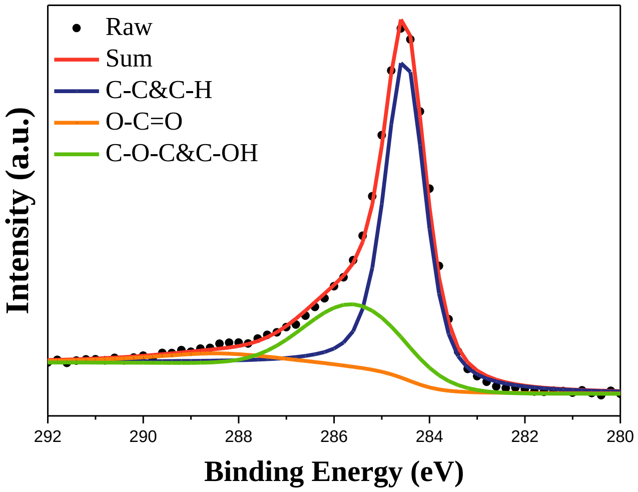

Figure S2. XPS spectra of γ-Fe_2O_3-Au-Ag/rGO.

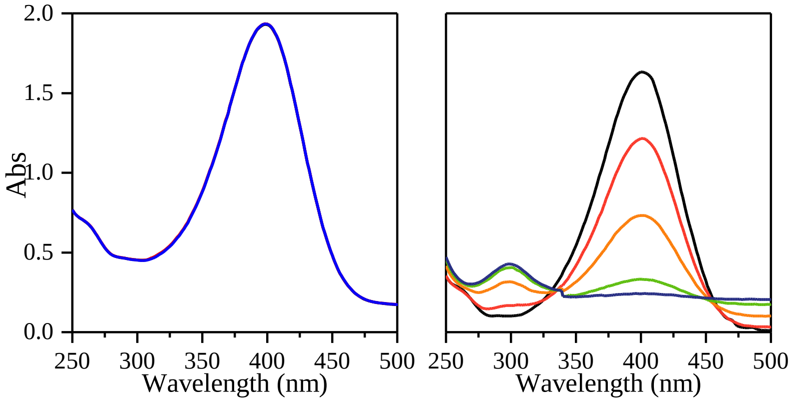

Figure S3. UV-vis absorption spectra of the reduction 4-NP by $NaBH_4$ in the presence of (**a**) AA-γ-Fe_2O_3/rGO, (**b**) AA-γ-Fe_2O_3-Au-Ag/rGO.

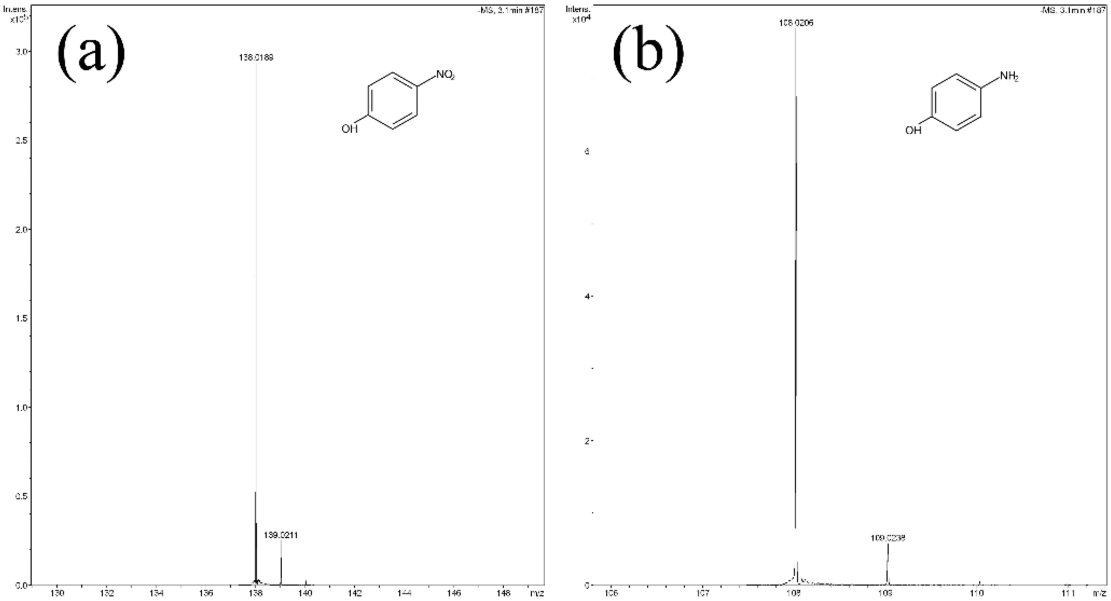

Figure S4. LC-MS spectrum for (**a**) 4-NP (**b**) for 4-AP (final product).

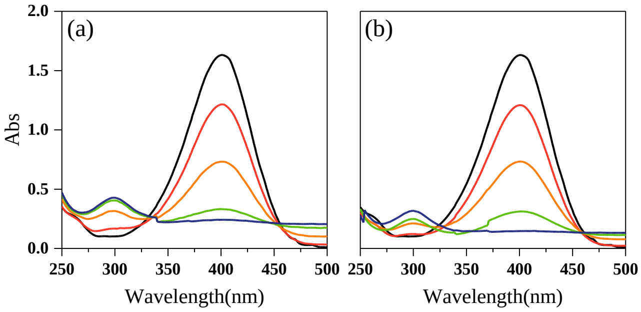

Figure S5. UV-vis absorption spectra of the reduction 4-NP by $NaBH_4$ in the presence of (**a**) AA-γ-Fe_2O_3-Au-Ag/rGO (**b**) AA-γ-Fe_2O_3-Au-Ag/rGO after running for five cycles.
